# Valproic Acid Combined with Zoledronate Enhance γδ T Cell-Mediated Cytotoxicity against Osteosarcoma Cells *via* the Accumulation of Mevalonate Pathway Intermediates

**DOI:** 10.3389/fimmu.2018.00377

**Published:** 2018-02-27

**Authors:** Shengdong Wang, Hengyuan Li, Chenyi Ye, Peng Lin, Binghao Li, Wei Zhang, Lingling Sun, Zhan Wang, Deting Xue, Wangsiyuan Teng, Xingzhi Zhou, Nong Lin, Zhaoming Ye

**Affiliations:** ^1^Department of Orthopedics, Centre for Orthopedic Research, Second Affiliated Hospital, School of Medicine, Orthopedics Research Institute, Zhejiang University, Hangzhou, China

**Keywords:** γδ T cells, zoledronate, histone deacetylase inhibitor, osteosarcoma, mevalonate pathway intermediates, synergism

## Abstract

The long-term survival of osteosarcoma has remained unchanged in the last several decades. Immunotherapy is proved to be a promising therapeutic strategy against osteosarcoma, especially for those with metastasis. Our previous study explored the sensibilization of zoledronate (ZOL) in γδ T cell-mediated cytotoxicity against osteosarcoma, but we have not yet elucidated the specific mechanism. Besides, high concentration is required to achieve these effects, whereas plasma ZOL concentration declines rapidly in the circulation. Valproic acid (VPA), a histone deacetylase inhibitor commonly used as the antiepileptic drug, has attracted much attention due to its synergistic antitumor efficacy with chemotherapy or immunotherapy. Here, we demonstrated that VPA combined with ZOL revealed the synergistic effect in enhancing antitumor efficacy of γδ T cells against osteosarcoma cells. This enhancement was mainly TCR-mediated and largely dependent on granule exocytose pathway. Of note, our findings indicated that ZOL sensitized osteosarcoma cells to γδ T cells by increasing the accumulation of the mevalonate pathway intermediates, which could be facilitated by VPA. We also found that this combination had similar effects on primary osteosarcoma cells. All the results suggested that VPA combined with ZOL could reduce the dose required to achieve a significant antitumor effect of γδ T cells, promoting it to be a novel therapy against osteosarcoma.

## Introduction

Osteosarcoma is the most common primary bone tumor that predominantly affects children and adolescents with a tendency for local invasion and pulmonary metastasis (1, 2). The long-term survival rate of patients with localized osteosarcoma has improved to 60–70% after the advent of multiagent chemotherapy regimens, but has reached a plateau over the last 30 years (3). Moreover, the 5-year overall survival rate of patients with relapsed disease or metastasis is less than 20% (4, 5). Therefore, the development of novel therapeutic strategies for osteosarcoma patients is urgently needed.

Immunotherapy has been considered to be an alternative strategy against malignancies, including osteosarcoma (6, 7). A variety of immune cells have been studied to treat osteosarcoma, such as natural killer (NK) cells, dendritic cells, macrophages, T cells, and so on (8–11). T cells play a critical role in mediating the antitumor immune response, and adoptive T cell therapy has been proposed as a promising option (12). Evidence suggested that cytotoxic T lymphocytes play a leading role in immune responses against tumor cells in osteosarcoma patients (13). It would be interesting to better precise that find an antigen specifically expressed on cancer cells and not in normal essential tissues, the real limit of CAR-T cells in solid tumors (14). γδ T cells, accounting for 1–10% of peripheral blood T cells (15), represent a potential candidate to kill tumor cells because of their direct recognition of tumor without the restriction of major histocompatibility complex (MHC) molecules (16). After recognizing tumor cells, activated γδ T cells will be able to directly kill target cells by engaging death receptors on the surface of tumor cells and producing cytotoxic granules and cytokines (17). Moreover, it has been verified that γδ T cells could effectively kill osteosarcoma cells both *in vitro* and *in vivo* (18, 19).

Zoledronate (ZOL), a third-generation aminobisphosphonate (ABP) already used in cancer patients, was reported to dramatically augment the cytotoxicity of γδ T cells against tumors (20–22). ZOL was found to inhibit farnesyl pyrophosphate synthase (FPPS) in tumor cells and increase the intracellular level of mevalonate pathway intermediates including isopentenyl pyrophosphate (IPP), which led to the activation of γδ T cells (23–25). In our previous study, we discovered the phenomenon that ZOL could sensitize osteosarcoma cells to the cytotoxicity of γδ T cells (26). Nevertheless, a general finding from the *in vitro* studies was that high concentrations of ZOL were required for antitumor effects, which had already exceeded those generally achievable *in vivo* (27, 28). Clinical tests have shown that plasma ZOL concentrations decline rapidly following an intravenous infusion, making it difficult to achieve and sustain high concentrations (29, 30). Consequently, we need to find some adjuvants to augment the effect of ZOL in order to reduce the required concentration of ZOL in inducing γδ T cell response against osteosarcoma.

Valproic acid (VPA), a well-known FDA approved histone deacetylase inhibitor (HDAC-I), is commonly used as an antiepileptic agent. VPA was shown to inhibit tumor proliferation and exert immunostimulatory activities *in vitro* and *in vivo* (31). Moreover, VPA showed promising capability in augmenting the anticancer efficacies of other therapeutic regimens, including ionizing radiation, chemotherapy, and immunotherapy (32, 33). Recently, it has been reported that VPA displayed antitumor activity against multiple kinds of malignant cells but exerted little cytotoxicity to normal cells (34). Besides, VPA was found to enhance the antitumor efficacy of immune cells by increasing the expression of NKG2D ligands (NKG2DLs) (35, 36). Interestingly, γδ T cells immunotherapy has been demonstrated to have more significant efficacy when in combination with chemotherapy or other strategies including HDAC-I (37). Recent study has revealed that VPA was related to the functional plasticity of γδ T cells (38). Taken together, we conjectured that VPA had the potential as an adjuvant to facilitate the antitumor activity of γδ T cells when combined with ZOL.

In the present study, we demonstrated the synergistic antitumor efficacy of γδ T cells against osteosarcoma cell lines in the presence of VPA and ZOL. In addition, we obtained similar effects on primary osteosarcoma cells. Furthermore, its mechanism we elucidated herein was ZOL induced mevalonate pathway blocking and intermediates accumulation, which could be enhanced by VPA. We further verified that γδ T cells cytotoxicity was mainly *via* TCR-mediated recognition and perforin pathway. Thus, our study confirmed the synergism of VPA and ZOL in inducing γδ T cells cytotoxicity and revealed a promising adoptive immunotherapy against osteosarcoma.

## Materials and Methods

### Ethical Statement

Research was approved by the Human Research Ethics Committees of the Second Affiliated Hospital, School of Medicine, Zhejiang University (Hangzhou, China). This research was performed in accordance with the Declaration of Helsinki and according to national and international guidelines. Written informed consent was obtained from all of the patients.

### Cell Line and Cell Culture

The human osteosarcoma lines HOS, U2OS, MG63, and Saos2 were obtained from Cell Bank of Shanghai Institute of Biochemistry and Cell Biology, Chinese Academy of Sciences (Shanghai, China). Their identity was verified by short tandem repeat analysis. The human primary osteosarcoma cells were derived from patients. HOS, MG63, Saos2, and primary cells were cultured in Dulbecco’s modified Eagle’s medium (Gibco, Rockville, MD, USA), U2OS cells in RPMI 1640 medium (Gibco), supplemented with 10% fetal bovine serum (Invitrogen, Carlsbad, CA, USA), and 100 µg/ml streptomycin–penicillin. Cells were maintained at 37°C in 5% CO_2_.

### γδ T Cell Expansion

Peripheral blood mononuclear cells, isolated from five healthy volunteers and seven patients with osteosarcoma by Ficoll gradient centrifugation, were cultured in RPMI 1,640 supplemented with 10% FBS and 1% penicillin/streptomycin. 1 µM zoledronate (Zometa; Novartis) and recombinant human IL-2 (400 IU/ml; PeproTech) were added at the first day, and then cells were supplemented with IL-2 at the same concentration every 3 days. Following 12–15 days culture, the cells were harvested and the purity of γδ T cells was determined by flow cytometry analysis. The γδ T cells can be further purified by magnetic activated cell sorting system (Miltenyi Biotech, Bergisch Gladbach, Germany). The percentage of γδ T cells from donors was more than 95% after purification (Figure S1 in Supplementary Material).

### Cytotoxic Assays and Blocking Studies

The cytotoxicity of human *ex vivo* expanded γδ T cells on osteosarcoma cells was evaluated by MTS assay. Osteosarcoma cell lines as well as primary tumor cells were seeded in 96-well plates at 3–5 × 10^3^ cells/well. After 24 h, they were pre-treated with VPA (Sigma) or/and ZOL at indicated concentrations for 24 h before co-cultured with γδ T cells from healthy volunteers or osteosarcoma patients at various E:T ratios. After co-culture for 2 h at 37°C, the supernatant was removed and the wells were softly washed with PBS twice. Then the cytotoxic effect was measured by MTS assay following the manufacturer’s instructions. A MR7000 microplate reader (Dynatech, NV, USA) was used to quantify the percentage of survived cells by determining the optical density. To inhibit mevalonate intermediates-mediated recognition by γδ T cells, osteosarcoma cells were treated with Mevastatin (Sigma) at 5 µM 1 h prior to treatment with VPA or/and ZOL for 24 h. Mevastatin was re-added at time of co-culture to maintain a constant concentration. To inhibit perforin-mediated cytotoxicity, γδ T cells were incubated with concanamycin A (CMA, Sigma) at 100 ng/ml for 2 h at 37°C before co-culture. To block the relevant cytotoxic pathways, specific mAbs were used at 10 mg/ml just before co-incubation assay.

### Antibodies and Flow Cytometry Analysis

Monoclonal antibodies specific for CD3, TCR-Vδ2, CD107a, IFN-γ,perforin were obtained from Biolegend (San Diego, CA, USA). For surface staining, the cells were incubated with the indicated fluorochrome-conjugated antibodies for 30 min in the dark at 4°C. After being washed twice, the cells were analyzed by flow cytometry according to the manufacturer’s instructions. γδ T cells degranulation was assessed by measuring the expression of CD107a according to the methods described elsewhere (39). In brief, γδ T cells were co-cultured with target cells for 2 h at 37°C in the presence of 10 µM monensin (Sigma). Then γδ T cells were harvested and stained with PE conjugated anti-Vδ2 and FITC-conjugated anti-CD107a. Intracellular staining of IFN-γ and perforin were performed to evaluate the cytokine production of γδ T cells. Briefly, γδ T cells were co-cultured with target cells for 2 h at 37°C in the presence of brefeldin A (20 µg/ml; BD Biosciences). Then γδ T cells were harvested and labeled with staining of anti-Vδ2 mAb before fixed and permeabilized using Cytofix/Cytoperm buffer (BD Pharmingen). After being washed twice in Perm/Wash buffer (BD Pharmingen), cells were stained with FITC-conjugated antihuman IFN-γ or FITC-conjugated antihuman perforin. Isotype-matched murine Xuorochrome-conjugated immunoglobulins from the corresponding manufacturer were used as negative controls. Flow cytometry was performed with FACSCanto (BD Biosciences) and data were analyzed using FlowJo software (Tree Star, San Carlos, CA, USA).

### Western Blot Analysis

Cells treated with VPA and ZOL for 24 h were centrifugated and lysed in RIPA buffer in the presence of proteasome inhibitor. Protein content was quantified by the BCA assay (Pierce, Rockford, IL, USA) according to the manufacturer’s instruction. Equal amounts of proteins (40 µg) were separated by SDS-PAGE and transferred onto polyvinylidene difluoride membrane (Millipore). The membrane were blocked with 5% bovine serum albumin in Tris-buffered saline with Tween 20 (TBST) for 2 h at room temperature and then incubated with primary antibodies at 4°C overnight. Blots were incubated with human anti-Ras (BD, San Jose, CA, USA), antiunprenylated Rap1A (Santa Cruz, CA, USA) and GAPDH (Cell Signaling Technology, Beverly, MA, USA) antibodies. After being washed with TBST, the membranes were incubated with an HRP-conjugated secondary antibody for 1 h at room temperature. Targeted bands were visualized using an enhanced chemiluminescence detection system (ChemiDoc™ XRS + imaging system; BIO-RAD, Hercules, CA, USA).

### Immunofluorescence

HOS and U2OS cells were seeded in a 12-well plates and treated with VPA and ZOL for 24 h. Then cells were fixed in 4% paraformaldehyde for 20 min at room temperature, permeabilized, and blocked for 30 min in 0.05% Triton X-100, and 2% bovine serum albumin. After being washed three times, fixed cells were incubated with antiunprenylated Rap1A overnight at 4°C. After that, cells were washed and incubated with a fluorescence-conjugated secondary antibody (Beyotime) for 2 h, and nuclei were stained with 4′,6-diamidino-2-phenylindole (KeyGen Biotech, Nanjing, China) for 3 min. Cells were observed with a fluorescence microscope (Leica).

### *In Vivo* Experiment

Healthy 4-week-old female BALB/c-nu mice were obtained from Experimental Animal Center of the Zhejiang Chinese Medical University and maintained under specific pathogen-free conditions and supplied with sterilized food and water. In the animal study, HOS cells were transfected with luciferase (HOS-Luc) and T cells were labeled with XenoLight DiR (Caliper life sciences, Hopkinton, MA, USA) with the purpose of *in vivo* imaging. For subcutaneous tumor model, HOS-Luc cells (5 × 10^6^ in 100 µl PBS) were injected subcutaneously into the right flank of each mouse. Mice were randomly separated into five groups (five mice each group). After 7 days, treatments of each group were set as follows: (1) untreated mice, receiving PBS, (2) γδ T cells (5 × 10^6^), (3) VPA (500 mg/kg) + γδ T cells (5 × 10^6^), (4) ZOL (50 µg/kg) + γδ T cells (5 × 10^6^), (5) VPA (500 mg/kg) + ZOL (50 µg/kg) + γδ T cells (5 × 10^6^). ZOL was administered intraperitoneally in 0.1-ml PBS twice a week and VPA was administered intraperitoneally in 0.1-ml PBS every 2 days. γδ T cells were administrated through tail vein the day after ZOL injection. Tumors were measured with caliper every 2 days and the tumor volume was estimated using the formula: volume = (length × width^2^)/2. The orthotopic bone tumor model was established according to the previous studies (40). A 30G needle was inserted to the proximal tibia through the cortex of the anterior tuberosity after the mouse anesthetized. Then HOS-Luc cells (1 × 10^6^ in 10 µl PBS) were injected slowly into the medullary cavity using Hamilton Syringe fitted with a 26G needle. In the orthotopic experiments, mice were also divided into five groups (four mice each group) with the same treatments as subcutaneous models. Mice in each group were imaged with an *In Vivo* Imaging System (Lumina Series III, Caliper life sciences). All treatments were performed for 2 weeks, and all mice were sacrificed by cervical dislocation after isoflurane inhalation. Tumors were dissected and stored in liquid nitrogen or fixed in formalin for further analysis. All experimental protocols were approved by the Animal Care and Use Committee of Zhejiang University, China.

### Immunohistochemical (IHC) Analysis

Formalin fixed and paraffin-embedded tumor specimens were cut into serial sections of 3 µm thickness. To show the intratumoral T cells, IHC staining of anti-CD3 (Abcam) was performed on consecutive tissue sections in accordance with previous studies (41). Images were obtained using a microscope.

### Statistical Analysis

All the data were analyzed using the SPSS software (version 16.0, SPSS, Chicago, IL, USA) and presented as mean ± SD. The statistical differences were detected by Student’s *t*-test, one-way analysis of variance (ANOVA) with Dunnett’s test or two-way ANOVA analysis. *p* < 0.05 was considered to be statistically significant.

## Results

### Combination of VPA and ZOL Showed Synergistic Effect of Enhancing γδ T Cell-Mediated Cytotoxicity against Osteosarcoma Cells

**Figure 1:**
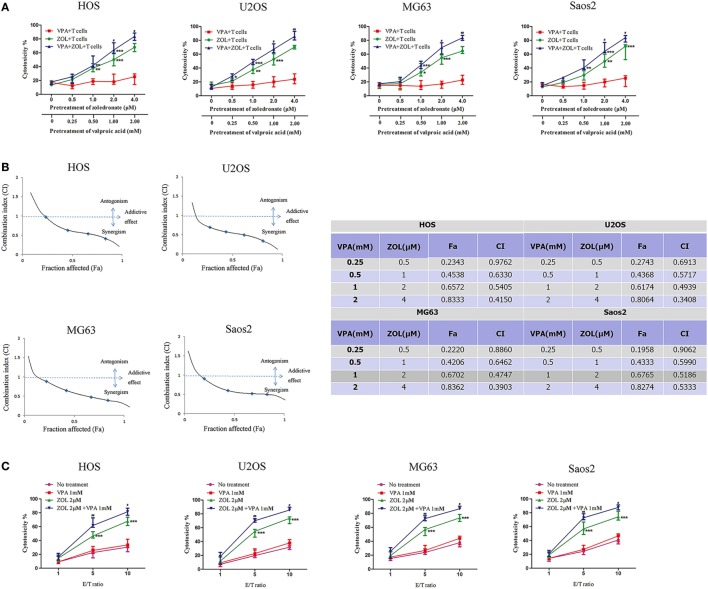
Valproic acid (VPA) and zoledronate (ZOL) synergistically induce γδ T cells cytotoxicity against osteosarcoma cells. **(A)** HOS, U2OS, MG63, and Saos2 cells were treated with different concentrations of VPA and ZOL at a certain ratio. After 24 h, tumor cells were co-cultured with γδ T cells at an E:T ratio of 5:1 for 2 h. Then the antitumor effect was measured by MTS assay. **(B)** The fraction affected (Fa) and combination index (CI) were calculated by Calcusyn. The synergistic effect of VPA and ZOL was determined by CI plot. **(C)** Tumor cells were pre-treated with control, 1 mM VPA, 2 µM ZOL, and VPA + ZOL, respectively, for 24 h. Then they were co-cultured with γδ T cells at the indicated E:T ratios for 2 h. The antitumor effect was measured by MTS assay. Each performed in triplicate. All the values were shown as mean ± SD; *p < 0.05, **p < 0.01, ***p < 0.001 versus no pre-treatment, ^#^*p* < 0.05,^##^ *p* < 0.01 versus pre-treatment with ZOL.

Treatment of HOS, U2OS MG63, and Saos2 cells with VPA or/and ZOL under a certain concentration did not show a significant cytotoxicity (Figure S2 in Supplementary Material). Tumor cells were exposed to various concentrations of VPA or/and ZOL for 24 h at a certain ratio before co-cultured with γδ T cells from healthy donors. MTS assay showed that γδ T cells displayed cytotoxicity against osteosarcoma cells pre-treated with ZOL or combination of VPA and ZOL in a dose-dependent manner (Figure 1A). Furthermore, combined pre-treatment showed significantly higher T cells cytotoxicity than ZOL alone, while there was little effect on untreated tumor cells or cells with VPA alone.

Combination index (CI) value, which was calculated by Calcusyn, was well accepted for quantifying drug synergism on the basis of multiple drug effect equation of Chou and Talalay (42, 43). A CI < 0.90 indicates synergism, a CI of 0.90–1.10 indicates an additive effect and a CI > 1.10 indicates antagonism. We calculated a series of CI values at different concentrations of VPA and ZOL. The synergistic effect was determined by CI plot (Figure 1B). Then we pre-treated the osteosarcoma cells with 1 mM VPA or/and 2 µM ZOL prior to co-cultured with γδ T cells at various E:T ratios. It showed significant synergism when E:T ratio reached 5:1 (Figure 1C).

### Combination of VPA and ZOL Increased the Level of γδ T Cells Cytotoxicity-Related Indicators against Osteosarcoma Cells

**Figure 2:**
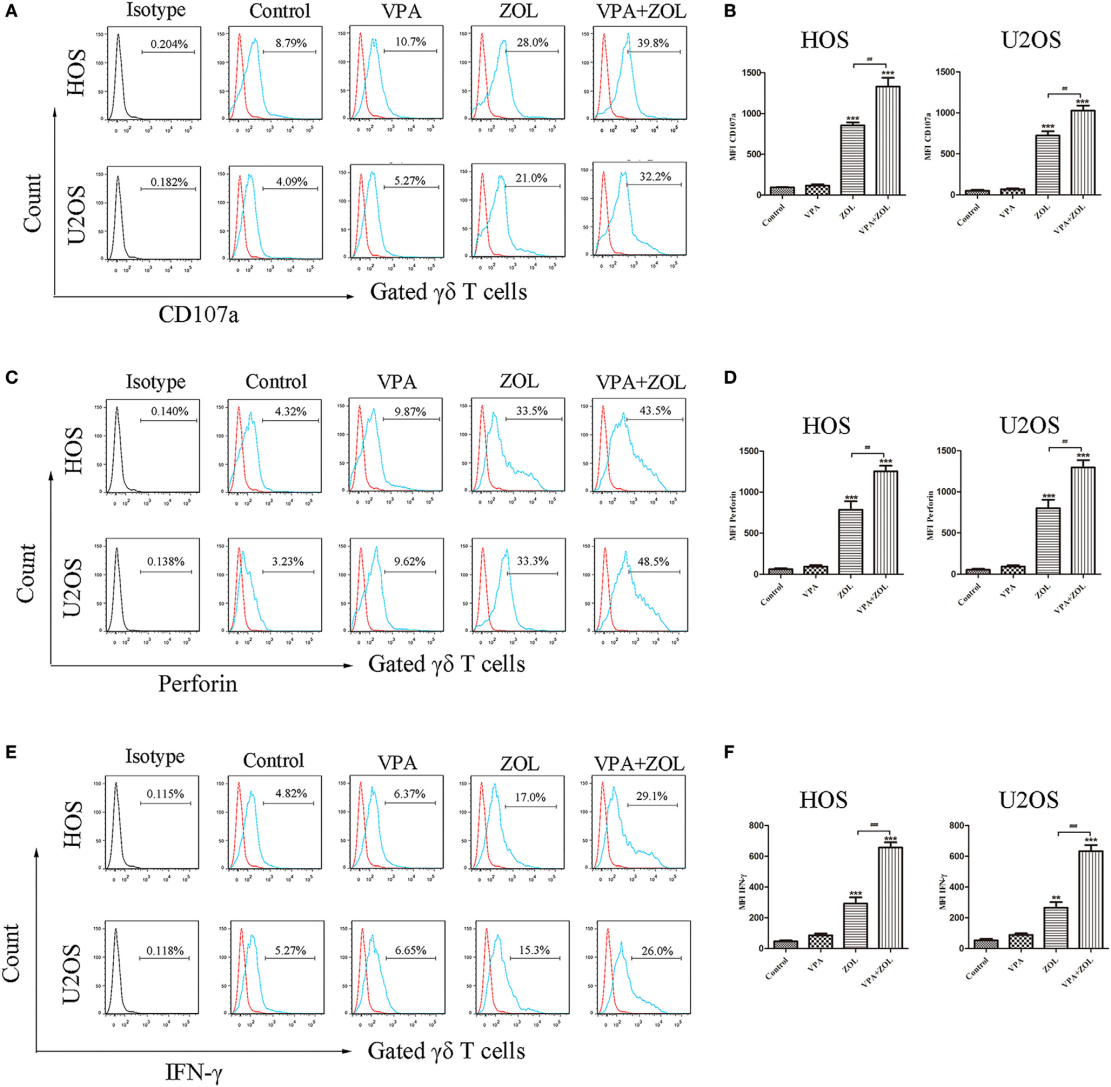
Valproic acid (VPA) and zoledronate (ZOL) upregulated the level of γδ T cells cytotoxicity-related indicators against osteosarcoma cells. HOS and U2OS cells were treated for 24 h with control, 1 mM VPA, 2 µM ZOL, and VPA + ZOL, respectively, before 2 h co-cultured with γδ T cells at an E:T ratio of 5:1. **(A,B)** CD107a expression levels on γδ T cells were measured by flow cytometry. MFI of CD107a^+^ γδ T cells was presented in histograms. **(C,D)** Perforin expression levels of γδ T cells were measured by flow cytometry. MFI of perforin^+^ γδ T cells was presented in histograms. **(E,F)** IFN-γ expression levels of γδ T cells were measured by flow cytometry. MFI of IFN-γ^+^ γδ T cells was presented in histograms. Red histogram line was isotype control and panels were overlapped. All the values were shown as mean ± SD from three independent experiments; *p < 0.05, **p < 0.01, ***p < 0.001 versus control, ^#^*p* < 0.05.

To clarify the mechanisms of γδ T cell-mediated cytotoxicity, we detected the expression of CD107a, perforin, and IFN-γ of γδ T cells co-cultured with HOS and U2OS cells after different pre-treatments for 24 h. The expression of CD107a, a degranulation marker of cytotoxic γδ T cells, was found to markedly rise when osteosarcoma cells were pre-treated with ZOL or VPA + ZOL (Figures 2A,B), while the level in the VPA pre-treatment group was almost the same as the untreated one. What’s more, combined treatments showed more significant effect of inducing the CD107a expression on γδ T cells. Similar results were obtained in the detection of perforin and IFN-γ of γδ T cells (Figures 2C–F). These results proved that treatment of VPA combined with ZOL could significantly enhance γδ T cells cytotoxicity in a synergistic way.

### VPA and ZOL Synergistically Sensitized γδ T Cells by Inducing the Accumulation of Mevalonate Pathway Intermediates due to FPPS Inhibition

**Figure 3:**
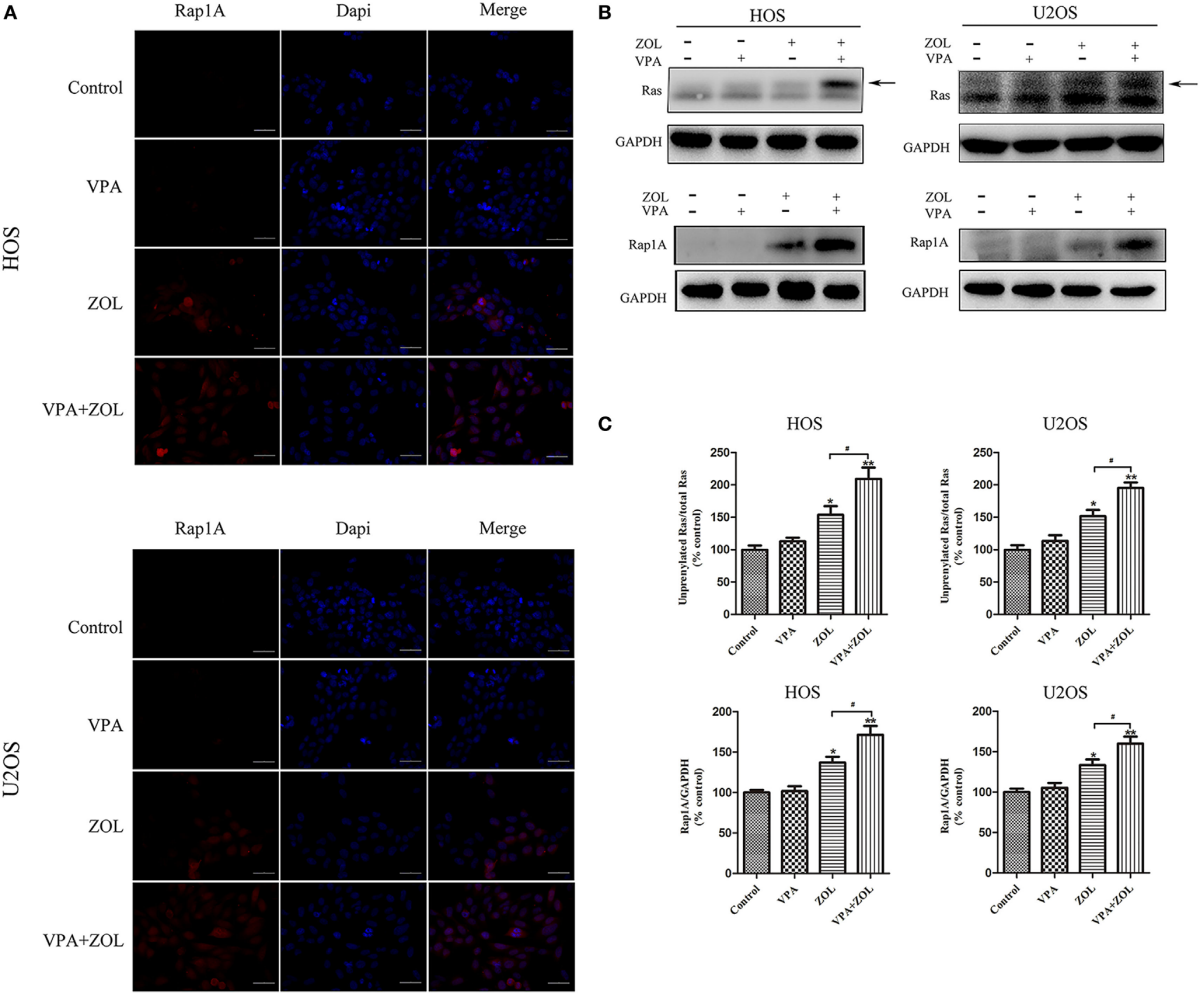
Valproic acid (VPA) and zoledronate (ZOL) inhibited prenylation of Ras and Rap1A in osteosarcoma cells. The expression levels of unprenylated proteins in HOS and U2OS cells with various treatments were detected by immunofluorescence and western blot. **(A)** The level of unprenylated Rap1A (red) was determined by fluorescence microscopy. The nuclei were counterstained with DAPI (blue). Scale bar: 50 µm. **(B,C)** The levels of unprenylated Rap1A and Ras were measured by western blot. The band marked by arrow corresponded to unprenylated form of Ras and the Rap1A antibody detected unprenylated form Rap1A. The levels of unprenyalted proteins were normalized and the relative values from three separate experiments were shown in histograms as mean ± SD; *p < 0.05, **p < 0.01 versus control, ^#^*p* < 0.05.

**Figure 4:**
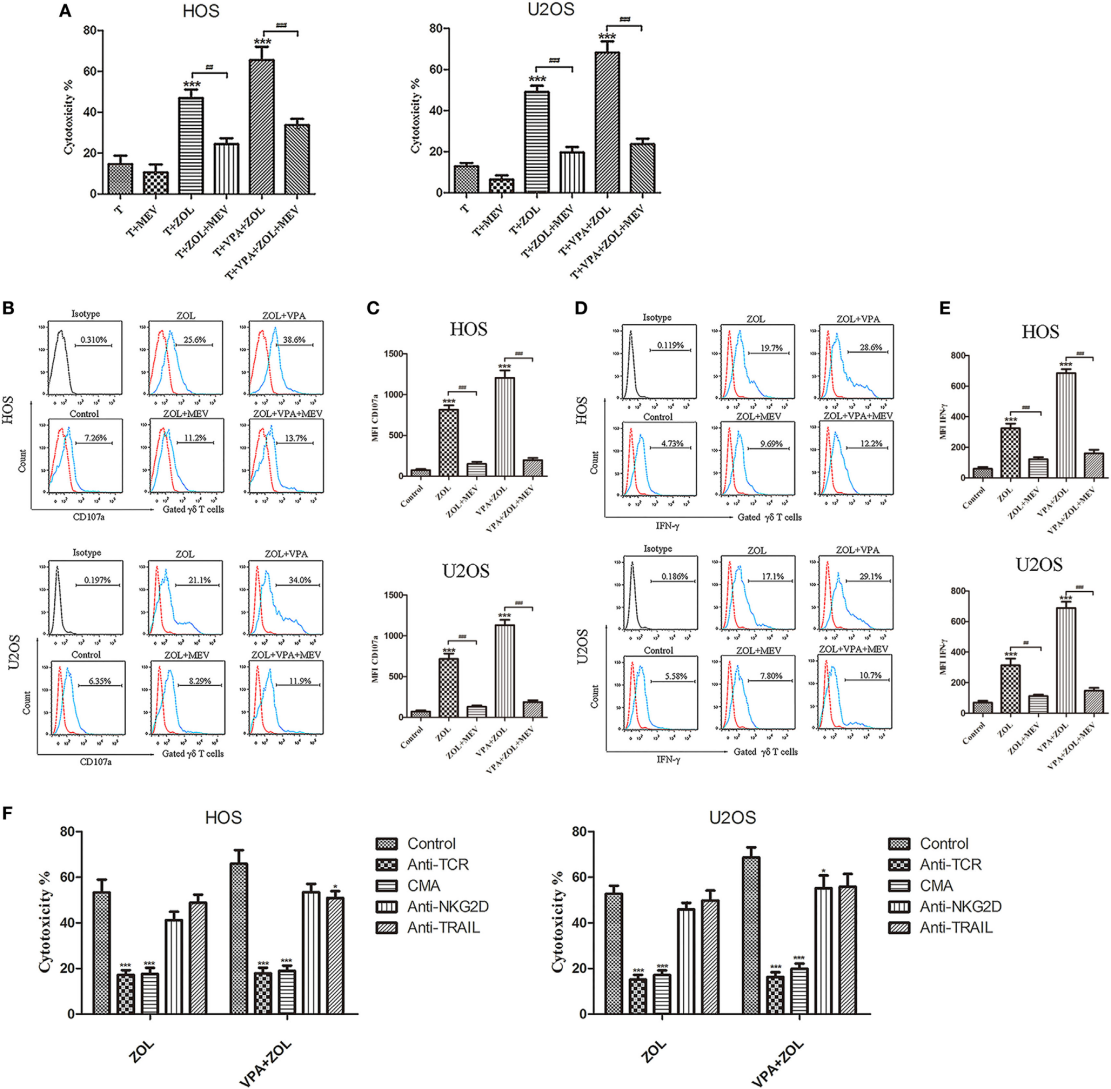
Mechanisms of the antitumor efficacy of γδ T cells induced by valproic acid (VPA) and zoledronate (ZOL). Osteosarcoma cells were pre-treated with 5 µM MEV 1 h prior to incubation with control or VPA or/and ZOL for 24 h. Then tumor cells were co-cultured with γδ T cells for 2 h at an E:T ratio of 5:1. **(A)** γδ T cells cytotoxicity against osteosarcoma cells was determined by MTS assay. The cytotoxic effect of γδ T cells induced by VPA and ZOL could be significantly inhibited by MEV **(B,C)** CD107a expression levels were evaluated by flow cytometry. MFI of CD107a^+^ γδ T cells was presented in histograms. **(D,E)** IFN-γ expression levels were evaluated by flow cytometry. MFI of IFN-γ^+^ γδ T cells was presented in histograms. **(F)** γδ T cells cytotoxicity was significantly inhibited in the presence of blocking Abs against TCR or concanamycin A (CMA), and partly reduced by blocking NKG2D or TRAIL. Red histogram line was isotype control and panels were overlapped. All the values were shown as mean ± SD from three separate experiments; *p < 0.05, ***p < 0.001 versus control [T in **(A)**], ^##^*p* < 0.01, ^###^*p* < 0.001.

Zoledronate was proved to increase the intracellular level of mevalonate pathway intermediates by inhibiting FPPS in some tumor cells, leading to γδ T cells sensitization. Previous studies showed that inhibition of FPPS resulted in the accumulation of unprenylated Ras and Rap1A (44, 45). The degrees of Ras and Rap1A prenylation were therefore used to determine FPPS blockade. In our experiments, HOS and U2OS cells treated with VPA and ZOL were shown to express significantly higher level of unprenylated Rap1A than ZOL alone in immunofluorescence, while tumor cells treated with VPA alone hardly express unprenylated Rap1A (Figure 3A). Besides, western blotting further showed that ZOL increased the expression of unprenylated Ras and Rap1A in HOS and U2OS cells significantly, and the combined treatment with VPA and ZOL exerted the stronger function (Figures 3B,C). Mevastatin, an inhibitor of hydroxy-methylglutaryl-CoA reductase blocking IPP synthesis (46), was used to reversely prove the mechanism. We found that the cytotoxicity of γδ T cells against HOS and U2OS cells treated with ZOL or VPA combined with ZOL significantly decreased in the presence of mevastatin (Figure 4A). Furthermore, mevastatin also obviously downregulated the level of CD107a and IFN-γ of γδ T cells sensitized by ZOL or combined treatment (Figures 4B–E). Therefore, it was suggested that increased levels of mevalonate pathway intermediates, such as IPP, seemed to be the ligands responsible for enhanced susceptibility of osteosarcoma cells to γδ T cell-mediated cytotoxicity.

### VPA and ZOL Induced γδ T Cells Antitumor Efficacy Mainly through TCR-Mediated Recognition and Perforin Pathway

γδ T cells were incubated with blocking Abs against TCR, perforin, NKG2D, and TRAIL in order to investigate the mechanism of γδ T cells antitumor efficacy induced by VPA and ZOL (Figure 4F). Either treated with ZOL or VPA + ZOL, cytotoxicity of osteosarcoma cells was inhibited at the greatest extent by anti-γδ TCR and CMA, indicating that the cytotoxic effect of γδ T cells was mainly mediated by TCR recognition and perforin pathway. NKG2D and TRAIL also seemed to play a minor role in mediating γδ T cells cytotoxicity.

### Combined Treatment with VPA and ZOL Further Induced γδ T Cells Migration and Enhanced γδ T Cells Antitumor Efficacy against Osteosarcoma *via* Prenylation Inhibition *In Vivo*

**Figure 5:**
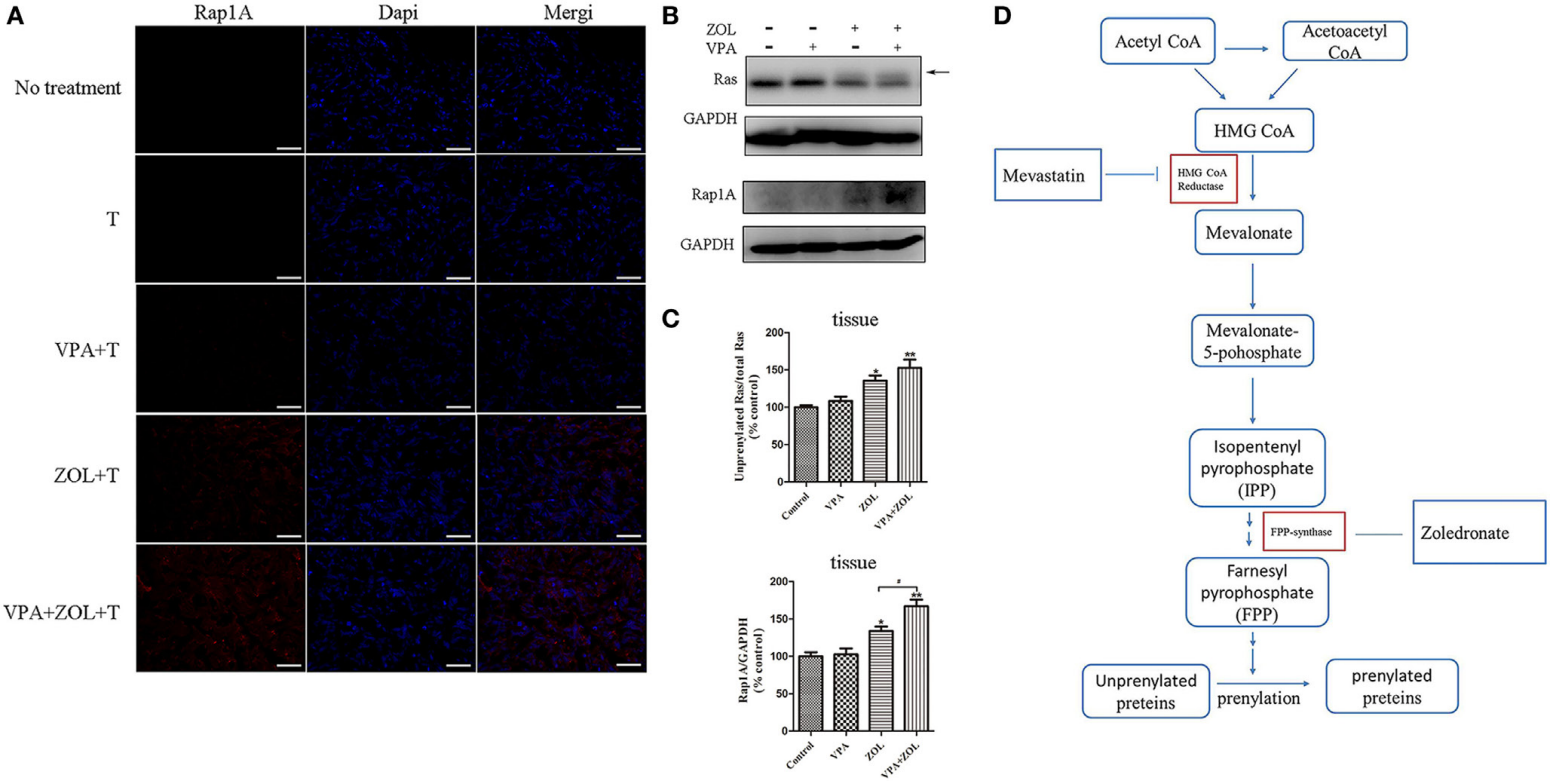
Inhibition of protein prenylation by *in vivo* treatment on osteosarcoma. Mice in different groups were sacrificed on 21th day after 2-week treatments and tumors were dissected. The expression levels of unprenylated proteins were detected by immunofluorescence and western blot. **(A)** The level of unprenylated Rap1A (red) was determined by fluorescence microscopy. The nuclei were counterstained with DAPI (blue). Scale bar: 50 µm. **(B,C)** The levels of unprenylated Rap1A and Ras were measured by western blot. The band marked by arrow corresponded to unprenylated form of Ras and the Rap1A antibody detected unprenylated form Rap1A. **(D)** Mevalonate pathway blockade induced by zoledronate (ZOL). The levels of unprenyalted proteins were normalized and the relative values were presented in histograms as mean ± SD; *p < 0.05, **p < 0.01 versus control, ^#^*p* < 0.05.

*In vivo* effect of VPA and ZOL to activate γδ T cells was determined in a tumor-transplanted mouse model. In subcutaneous models, tumor tissues in ZOL or VPA + ZOL treated BALB/c-nu mice showed marked unprenylated Rap1A and Ras expression, which were demonstrated in immunofluorescence and western blotting (Figures 5A–C), while these unprenylated proteins were hardly detected in VPA-treated or untreated xenografts. Similar to the results of *in vitro* studies, the levels of unprenylated proteins expression were shown to be significantly higher in the combined treatment group than that with ZOL alone. The way of FPPS inhibited by ZOL in mevalonate pathway in osteosarcoma cells was shown in Figure 5D.

**Figure 6:**
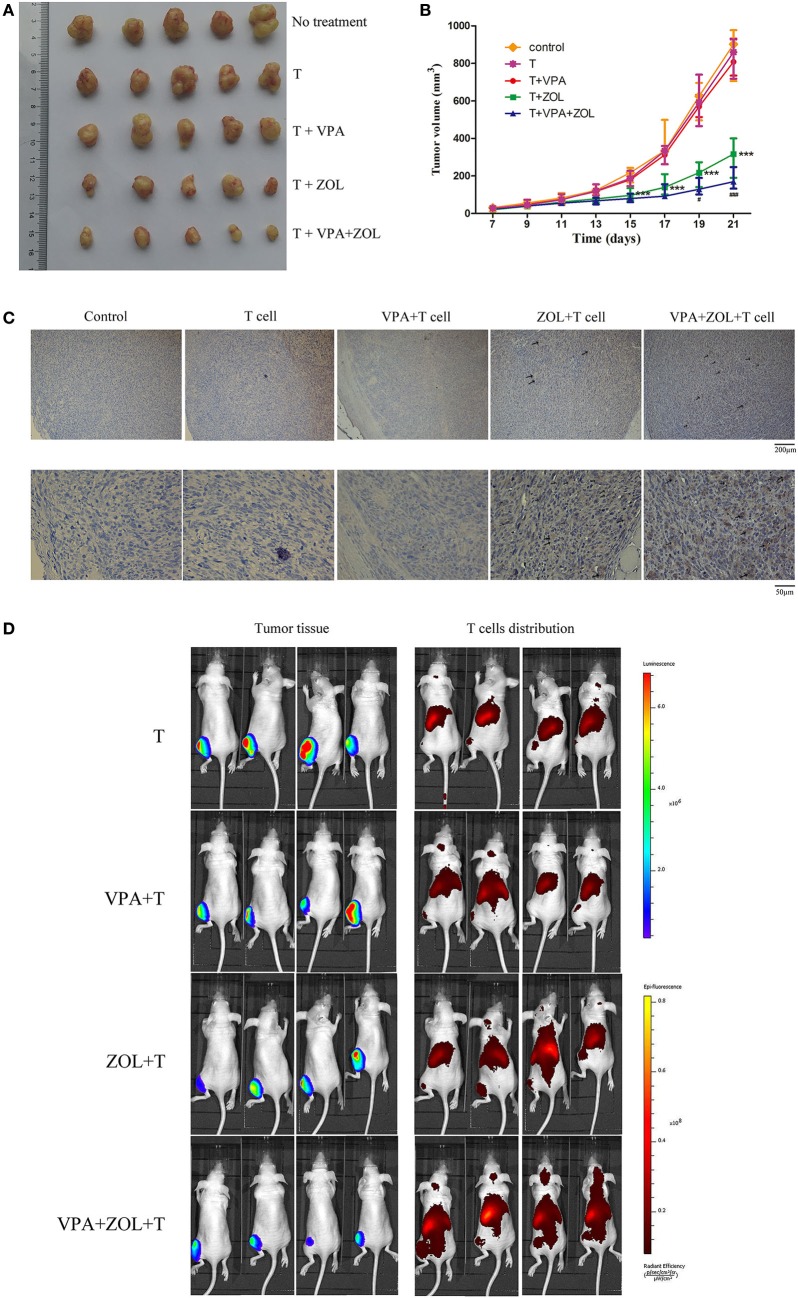
Valproic acid (VPA) and zoledronate (ZOL) enhanced γδ T cells cytotoxicity against osteosarcoma in xenograft models. HOS cells transfected with luciferase (HOS-Luc) were inoculated subcutaneously into the right flank of BALB/c-nu mice. After 7 days, mice started to receive various treatments and injection of γδ T cells. **(A)** Tumor excised from mice on 21th day. **(B)** Tumor volumes were measured every 2 days, starting on the seventh day. **(C)** Intratumoral γδ T cells were detected by immunohistochemical assays (shown in brown and indicated with arrowheads). **(D)** Orthotopic models were established using HOS-Luc cells and BALB/c-nu mice. After 7 days, mice started to receive various treatments and injection of γδ T cells. Mice were imaged with *in vivo* imaging system 24 h postinjection of γδ T cells. Tumor growth was evaluated by visualizing bioluminescence and T cells migration was shown by DiR fluorescence. All the values were shown as mean ± SD; ***p < 0.001 versus control, ^#^*p* < 0.05, ^###^*p* < 0.001 versus treatment with ZOL and γδ T cells injection.

As shown in Figures 6A,B, γδ T cells only induced limited tumor growth inhibition in VPA-treated or untreated mice but exerted significant antitumor efficacy in the group with ZOL and VPA + ZOL. In the combined pre-treatment group, VPA and ZOL showed further enhancement in activating γδ T cells. On the 21th day, the mean tumor volume in mice without any treatment was 876.6 mm^3^ (706–977 mm^3^), and it was 827.0 mm^3^ in mice treated with T cells alone (717–927 mm^3^), 824.0 mm^3^ in mice with T cells and VPA (734–931 mm^3^), 310.5 mm^3^ in mice with T cells and ZOL (190–387 mm^3^) and 177.6 mm^3^ in mice with T cells and VPA combined with ZOL (132–247 mm^3^). It was also shown that VPA and ZOL could further induce γδ T cells migration. Intratumoral γδ T cells were detected by IHC assays using CD3 antibody. In our studies, we found numerous T cells within tumor tissue in mice treated with ZOL and significantly more in mice received VPA and ZOL, whereas few T cells were detected in the other groups (Figure 6C).

To further display T cell distribution and antitumor efficacy, *in vivo* imaging system was utilized in the orthotopic bone tumor model to detect bioluminescence by luciferase-transfected tumor tissue and the fluorescence by DiR labeled T cells. As shown in Figure 6D, in mice pre-treated with ZOL, tumor growth was significantly inhibited and the injected γδ T cells clustered at the tumor site. And similarly, stronger effects were shown in the group with combined treatments. But in mice with VPA or no treatment, tumors were relatively larger and γδ T cells were mainly distributed in the liver and spleen. All these results indicated that VPA and ZOL have the potential to synergistically strengthen γδ T cell-mediated cytotoxicity against osteosarcoma.

### VPA and ZOL Had the Similar Effects on Primary Cells

**Figure 7:**
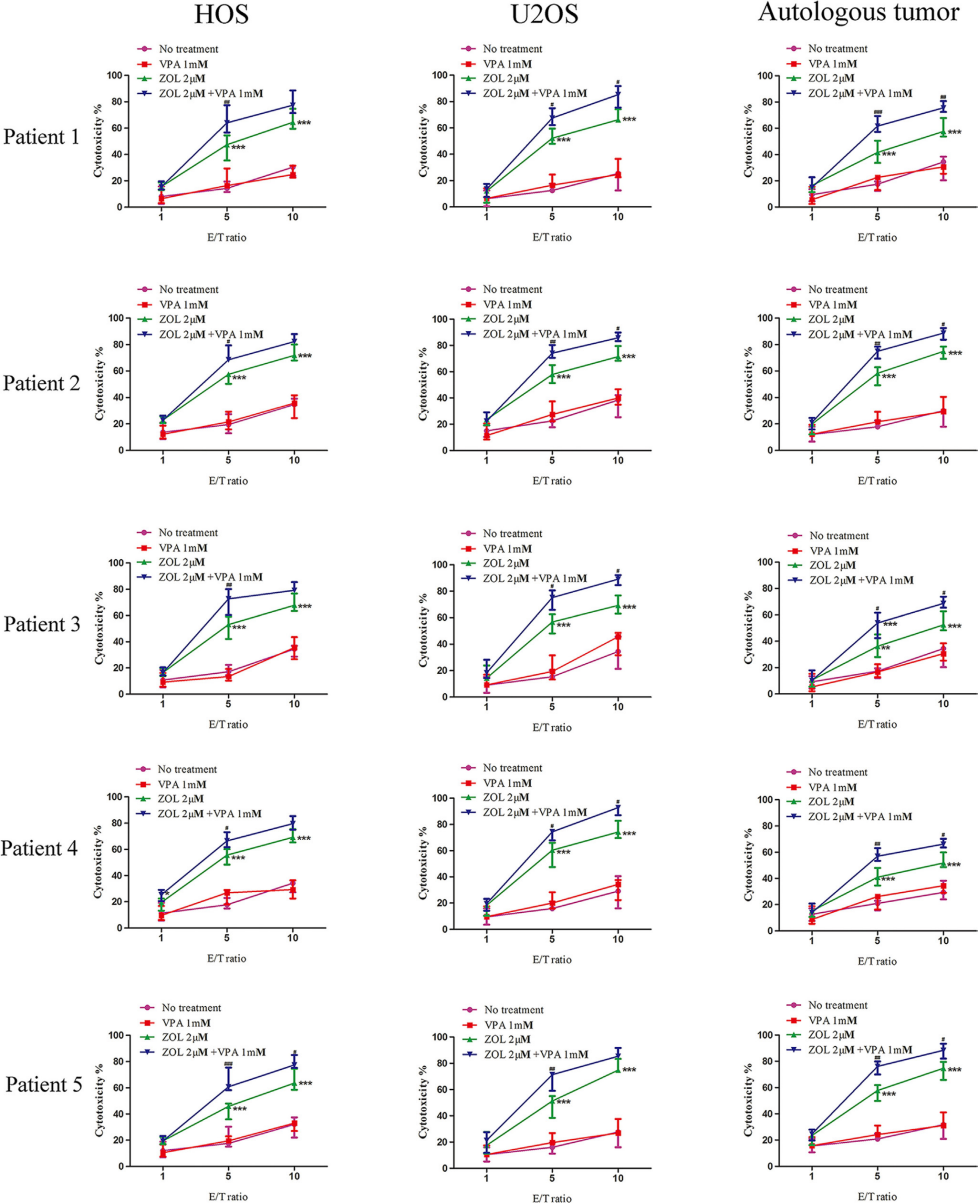

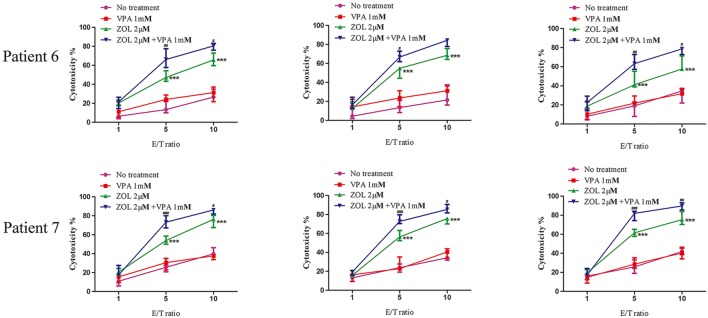
Valproic acid and zoledronate (ZOL) enhanced γδ T cells cytotoxicity against osteosarcoma cells in primary cell experiment. HOS and U2OS cells as well as autologous tumor cells were treated with various treatments for 24 h before co-cultured with γδ T cells derived from osteosarcoma patients at the indicated E:T ratios. Cytotoxicity of γδ T cells against tumor cells was evaluated by MTS assay. All the values were shown as mean ± SD; *p < 0.05, **p < 0.01, ***p < 0.001 versus no pre-treatment, ^#^*p* < 0.05,^##^ *p* < 0.01, ^###^*p* < 0.001 versus pre-treatment with ZOL.

We obtained primary osteosarcoma cells and expanded γδ T cells from seven patients suffered from osteosarcoma to demonstrate the synergistic effects of VPA and ZOL on clinical specimens. The information of specimens is shown in Table S1 in Supplementary Material. As expected, primary γδ T cells exerted similar cytotoxicity against osteosarcoma cell lines as well as autologous tumor cells. Only osteosarcoma cells pre-treated with ZOL or VPA + ZOL induced significant antitumor effect of γδ T cells, and enhanced cytotoxicity was shown in combined treatment (Figure 7). These data reveal that VPA and ZOL have similar synergistic effects on primary cells.

## Discussion

Osteosarcoma is the second cause of cancer-related death in adolescents (47). Despite advances in surgery and multiagent chemotherapy, the prognosis of this disease is still unsatisfactory due to inefficient response to drug therapy. Immunotherapy is considered to be a promising therapeutic strategy against osteosarcoma, especially in adjuvant therapy. Some studies revealed that osteosarcoma cells were potentially susceptible to NK cells lysis in an NKG2D-NKG2DL manner (48, 49). Studies on NK-related cytotoxicity against osteosarcoma have been conducting all the time (48, 50). Numerous significant achievements have been made in T cell-based immunotherapy for osteosarcoma (11), which seems to be the most promising therapeutic strategy. Insufficient level of tumor associated antigen expression is the main obstacle to limit cytotoxic T cells efficacy against osteosarcoma and some other malignant diseases. Our previous studies have demonstrated that CD8^+^ T cells could specifically kill osteosarcoma cells due to the upregulation of cancer/testis antigens following treatment with demethylating agent *in vitro* and *in vivo*, but this effect was MHC-restricted (51). Moreover, we have also proved that osteosarcoma cells were highly susceptible to the cytotoxicity of γδ T cells in the presence of ZOL (26). Nevertheless, it is difficult to keep high-plasma concentration ZOL as a result of rapid degradation. It seems that combination of ZOL with another adjuvant may be a promising therapy to strengthen the γδ T cells cytotoxicity.

Histone deacetylase inhibitor is considered to be one of the most effective anticancer agents (52, 53). VPA, trichostatin A, and suberoylanilide hydroxamic acid have already been used in clinical trials (54). VPA was proved to enhance the efficacy of chemotherapy and immunotherapy with little impact on normal cells so that it was used as an adjuvant in this experiment. Our present study confirmed that the cytotoxicity of γδ T cells against osteosarcoma cells in the presence of ZOL could be significantly enhanced by VPA. Then we verified the synergistic effect of VPA and ZOL using CI, which is used to determine the synergism and antagonism in drug combination study (55). Subsequently, we further detected some effective antitumor-related indicators of γδ T cells to confirm the synergistic effect, including CD107a, perforin, and IFN-γ. All these indicators correlated with the antitumor capacity of T cell (56–58). Besides, the cytotoxic effect of γδ T cells was proved mainly *via* TCR and perforin pathway. In studies on mice, γδ T cells exerted the most effective control of the tumor growth in the group pre-treated with VPA + ZOL. We also found more concentrated distribution of T cells around tumor site in the combination group. To verify whether this synergistic effect applies to clinical specimens, we collected γδ T cells and primary tumor cells from patients of osteosarcoma. As expected, we found VPA and ZOL had similar effects on specimens from osteosarcoma patients. All the results showed that VPA combined with ZOL could significantly enhance the γδ T cell-mediated antitumor efficacy against osteosarcoma cells.

It has been demonstrated that ZOL could inhibit FPPS and upregulate the level of IPP, which is the stimulatory antigen for γδ T cells (59, 60). Interestingly, IPP accumulation is less efficient in non-transformed cells than tumor cells with a pharmacologically relevant concentration of ABP (45, 61), allowing the immunotherapy for cancer by activating γδ T cells with ABP such as ZOL. Some GTP-binding proteins, such as Ras and Rap1A, are used to indirectly detect the mevalonate pathway blocking and accumulation of intermediates like IPP (62, 63). In our study, we proved that ZOL induced accumulation of mevalonate pathway intermediates in osteosarcoma cells, which could be significantly facilitated by VPA. That seems to be the internal mechanism of the synergistic effect of VPA and ZOL in inducing γδ T cells cytotoxicity against osteosarcoma cells.

Some chemotherapeutic drugs are proved to improve the effect of immunotherapy, whereas most of them have severe side effects and partial response. Specifically, both VPA and ZOL have been used in clinic for years, and little toxicity was detected at the dose we used in this research, which ensure the safety of clinical application. Furthermore, our study confirmed the efficient expansion of γδ T cells from osteosarcoma patients and their effective cytotoxicity against autologous tumor cells, which indicated the potential adoptive therapy using patient-derived γδ T cells with the combination of VPA and ZOL.

In conclusion, our study is the first to demonstrate that VPA combined with ZOL could synergistically induce γδ T cell to kill osteosarcoma cells by enhancing accumulation of mevalonate pathway intermediates. Similar effects are also shown in xenograft model and primary cells. These compelling evidences help us better understand the mechanism of γδ T cells-related cytotoxicity and contribute to the clinical adoptive γδ T cells immunotherapy.

## Ethics Statement

Research was approved by the Human Research Ethics Committees of the Second Affiliated Hospital, School of Medicine, Zhejiang University (Hangzhou, China). This research was performed in accordance with the Declaration of Helsinki and according to national and international guidelines. Written informed consent was obtained from all of the patients.

## Author Contributions

SW, HL, and ZY designed the study and analyzed the data. SW, HL, CY, PL, BL, and ZY performed the experiments. SW, WZ, and ZY wrote the manuscript. All authors read and approve the final manuscript. SW and HL were co-first authors.

## Conflict of Interest Statement

The authors declare that the research was conducted in the absence of any commercial or financial relationships that could be construed as a potential conflict of interest.

## Supplementary Material

Figure S1 *Ex vivo* expansion of γδ T cells. Representative flow cytometry of γδ T cells from volunteers after 14 days culture and magnetic beads separation.

Figure S2 Valproic acid (VPA) and/or zoledronate (ZOL) under a certain concentration showed little cytotoxicity on four osteosarcoma cell lines. HOS, U2OS, MG63, and Saos2 cells were treated for 24 h with different concentrations of VPA and ZOL at a certain ratio. Then the cell viability was measured by MTS assay.
