# Supplementary figures and images for: Valproic Acid Combined with Zoledronate Enhance γδ T Cell-Mediated Cytotoxicity against Osteosarcoma Cells via the Accumulation of Mevalonate Pathway Intermediates
Source: Front Immunol. 2018 Feb 27;9:377. doi: 10.3389/fimmu.2018.00377 (PMC5835048; doi:10.3389/fimmu.2018.00377)

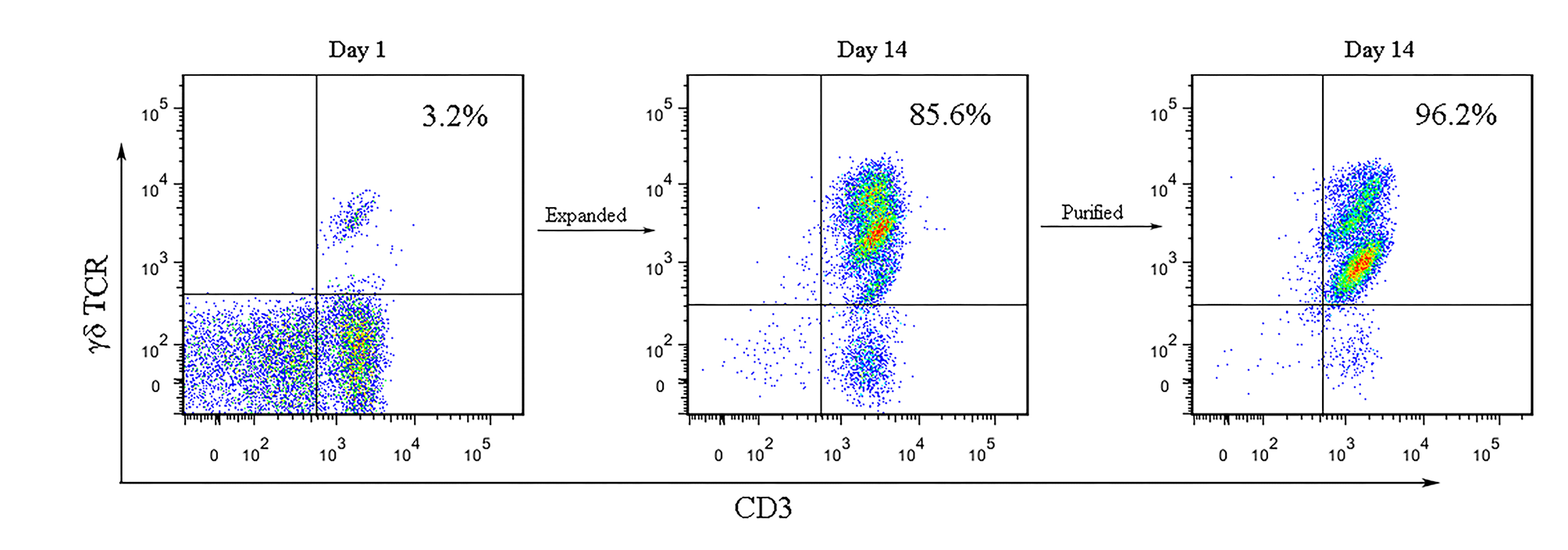

Supplement: Figure S1 — Ex vivo expansion of γδ T cells. Representative flow cytometry of γδ T cells from volunteers after 14 days culture and magnetic beads separation. [file Image_1.tif]

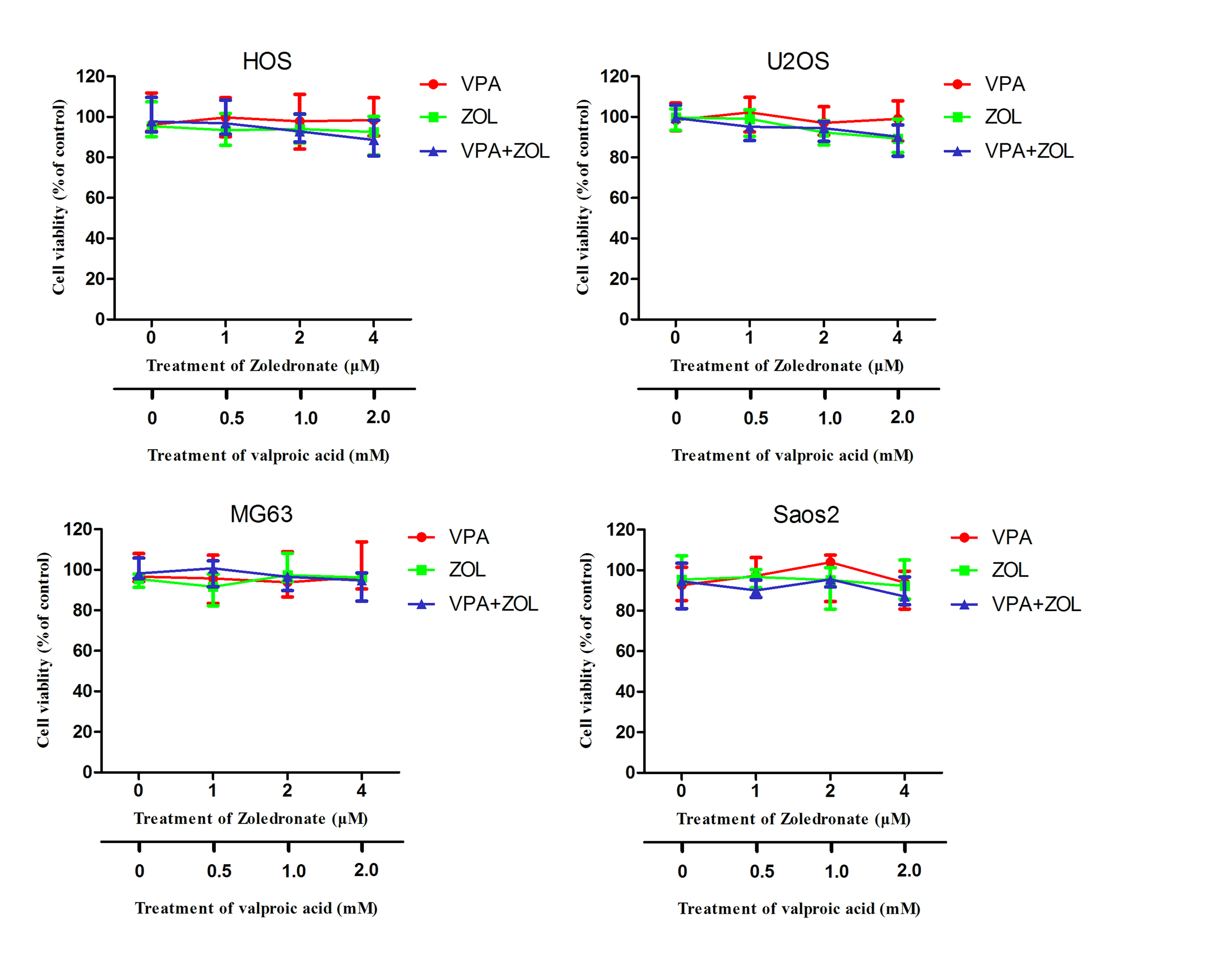

Supplement: Figure S2 — Valproic acid (VPA) and/or zoledronate (ZOL) under a certain concentration showed little cytotoxicity on four osteosarcoma cell lines. HOS, U2OS, MG63, and Saos2 cells were treated for 24 h with different concentrations of VPA and ZOL at a certain ratio. Then the cell viability was measured by MTS assay. [file Image_2.tif]
